# Maternal Supplementation with *Lacticaseibacillus rhamnosus* GG Improves Glucose Tolerance and Modulates the Intestinal Microbiota of Offspring

**DOI:** 10.3390/diseases12120312

**Published:** 2024-12-03

**Authors:** Dayane Correia Gomes, José Enrique Meza Alvarado, Jesus Alejandro Zamora Briseño, Cynthia Cano Sarmiento, Alberto Camacho Morales, Rubi Viveros Contreras

**Affiliations:** 1 Centro de Investigaciones Biomédicas, Doctorado en Ciencias Biomédicas, Universidad Veracruzana, Xalapa 91190, Mexico; dayanecorreiagomesbio@gmail.com (D.C.G.); enmeza@uv.mx (J.E.M.A.); 2 Advanced Molecular Studies Network, Campus III, Institute of Ecology A. C., Xalapa 91612, Mexico; alejandro.zamora@inecol.mx; 3 Food Research and Development Unit, Technological Institute of Veracruz, National Institute of Technology of Mexico, M.A. de Quevedo 2779, Veracruz 91897, Mexico; cynthia.cs@veracruz.tecnm.mx; 4 Faculty of Medicine, Department of Biochemistry and Molecular Medicine, Autonomous University of Nuevo León, Monterrey 66455, Mexico; alberto.camachomr@uanl.edu.mx

**Keywords:** *Lacticaseibacillus rhamnosus*, maternal programming, metabolic syndrome, glucose intolerance, high-fat diet

## Abstract

Introduction: Consuming hypercaloric diets during pregnancy induces metabolic, immune, and maternal intestinal dysbiosis disorders. These conditions are transferred to the offspring through the placenta and breastfeeding, increasing susceptibility to metabolic diseases. We investigated the effect of *L. rhamnosus* GG supplementation on offspring maternally programmed with a hypercaloric diet. Methods: Our study involved sixteen female Wistar rats aged ten weeks, which were divided into four groups based on their diets: control (Ctrl), cafeteria (CAF), control + probiotic (PRO), and cafeteria + probiotic (CPRO). The control + probiotic and cafeteria + probiotic groups received a daily oral administration of 250 μL of *L. rhamnosus* GG cell suspension (equivalent to 10^9^ UFC) for nine weeks. The body weight of the animals was recorded weekly, and their food intake was monitored every 24 h. An oral glucose tolerance test was conducted on the offspring at seven weeks of age. At the ninth week of age, animals were euthanized, and blood, tissues, and organs were collected. Results: Maternal supplementation with *L. rhamnosus* GG decreased food intake and the average birth weight, improved glucose sensitivity, and lowered the levels of LDL, cholesterol, triglycerides, and mesenteric adipose tissue in offspring compared with the control and cafeteria groups. Conclusions: Our findings indicate that supplementing with LGG during maternal programming could protect offspring from metabolic disruptions caused by a hypercaloric maternal diet.

## 1. Introduction

Maternal programming is a physiological phenomenon that combines exposure of the intrauterine environment to external or genetic stimuli or factors and implies that factors in the intrauterine environment can affect the pre-and postnatal health of the offspring [1]. Thus, exposure of the fetus and newborn to negative external stimuli can impact the offspring’s structural, physiological, and metabolic functions, which can hinder the optimal development of the offspring both pre- and postnatally [2,3]. For these reasons, negative environmental stimuli or manipulations at critical stages of pregnancy (e.g., first trimester) can cause damage to fetal growth, compromising the development of fetal health and causing alterations in embryonic and fetal growth homeostasis [4,5].

Maternal nutrition is a crucial element that can have beneficial or adverse effects on fetal programming, depending on its nutritional contributions [6]. For example, maternal diet and nutritional compositions influence the development of epigenetic profiles in the fetus. Some of these epigenetic changes are long lasting and may significantly affect the offspring’s metabolism [7,8]. In addition, hypercaloric maternal diets high in fat and sugars can cause placental dysfunction and limited intrauterine fetal growth, which can prevent higher birth weights and contribute to long-term health consequences [9,10]. Likewise, hypercaloric maternal diets high in fat and sugars can cause placental dysfunction and limited intrauterine fetal growth, which can result in premature neonates with meager birth weights and contribute to short and long-term health consequences [7].

The gut microbiota is a complex ecosystem of microorganisms living in the gastrointestinal tract, and it affects various physiological processes, such as the immune, metabolic, and nutritional systems. The gut microbiota plays a vital role in the development of the fetus during pregnancy [11,12,13] because the placenta harbors a diverse microbial community that is important in forming the fetal gut microbiota. In this way, the maternal diet, immune system, and hormonal changes during pregnancy can contribute to the transfer of maternal gut microbiota to the fetus via the placenta [11,13]. There are reports that inadequate feeding during pregnancy induces maternal intestinal dysbiosis (e.g., imbalance of the intestinal microbial profile). This phenomenon can cause immune imbalance, disruption of the intestinal barrier and transmission of bacteria to the intrauterine cavity, placental inflammation, and poor placentation, which can negatively impact the growth and neurodevelopment of the fetus, promoting immunological and metabolic alterations in the offspring [12,14,15].

Probiotic supplementation during maternal programming can improve maternal plasma glucose control, reduce the development of eczema in infants, and decrease pregnancy complications such as preterm delivery, preeclampsia, and gestational diabetes [16]. In this context, it has been reported that the strain *Lacticaseibacillus rhamnosus* GG (LGG) can impact body weight and body fat and reduce cholesterol and triglyceride levels in male mice fed a hypercaloric diet [17]. The current study investigated how supplementation with *Lacticaseibacillus rhamnosus* GG during maternal programming on a high-calorie diet affects the offspring’s metabolic health and gut microbiota.

## 2. Materials and Methods

### 2.1. Animal Handling

Programming and mating experiments were performed using sixteen virgin female rats and eight male Wistar strains of 8 to 10 weeks of age, respectively. The animals were handled according to the NOM-ZOO-1999 guide for the care and use of laboratory animals after approval by the Institutional Committee for the Care and Use of Laboratory Animals (CICUAL) of the Health Sciences Unit (HSU) with registration number CICUAL-UCS-07.

### 2.2. Preparation of Bacterial Culture

The *Lacticaseibacillus rhamnosus* GG 53103TM strain (isolated by Gorbach and Goldin) was purchased from ATCC in Mexico City, Mexico. The *L. rhamnosus* GG was cultured in Man Rogosa Sharp (MRS) broth medium (Merck Millipore, Burlington, MA, USA) at 37 °C for 24 h. Bacterial cells were pelleted at 4000× *g* (4 °C) for 10 min and were resuspended in a phosphate buffer (Sigma-Aldrich, St. Louis, MO, USA) at a final concentration of 10^9^ CFU/mL.

### 2.3. Maternal Nutritional Programming Model

The animals were divided into pairs (i.e., two per cage) and kept in polycarbonate cages at 22 °C with a 12/12 h light–dark photoperiod. After six days, the female rats were divided into four experimental groups according to dietary intervention: control (Ctrl, *n* = 4), cafeteria (CAF, *n* = 4), control + probiotic (PRO, *n* = 4), and cafeteria + probiotic (CPRO, *n* = 4). The intervention lasted nine weeks (considering three weeks of pre-pregnancy, three weeks of gestation, and three weeks of lactation). The animals had ad libitum access to water during this period. For the control diet, we used a standard diet for rodents, containing 10% kcal of fat with a calorie density of 3.35 kcal·g^−1^ divided into 71% carbohydrates, 11% lipids, and 18% proteins (Research Diets, New Brunswick, NJ, USA, Cat. D12450B). The CAF diet included liquid chocolate, biscuits, fried potatoes, smoked pork bacon, standard diet control, and pork paté in a 1:1:1:1:1:2 ratio, respectively. This diet had a calorie density of 3.72 kcal·g^−1^, containing 39% carbohydrates, 49% lipids, and 12% proteins [18].

Every day, *L. rhamnosus* GG (LGG) was administered to female rats in the PRO and CPRO groups via oral gavage at 250 µL of cell suspension (final concentration of 10^9^ CFU/mL). The control and CAF groups were administered sterile PBS (phosphate-buffered saline) via the same procedure [19].

For the mating stage, the female rats were housed in pairs, and a male rat (weighing 300 to 350 g) was placed per couple for 48 h. Then, the male rats were removed from the female cages. Pregnancy was confirmed by the presence of a vaginal cap the next day, considering day 0.5 of pregnancy. After reproduction, the females were individually lodged and continued with their assigned diet. We evaluated the uniformity of ten offspring, including males and females from each mother in every group. Male offspring exposed to different maternal diets were weaned at day 21, grouped into eight subjects per group, and assigned to four groups according to the maternally programmed diet. They were maintained under standard rodent diet feeding.

### 2.4. Body Weight, Food Intake, and Energy Efficiency

The weight of the female mice (mothers) was monitored from pregnancy until the birth of their offspring; after the birth of the male offspring, the weight of each was recorded weekly using a digital scale until two months of age (every eight days), using a digital scale following standardized procedures. Feed consumption was evaluated by subtracting the amount of feed provided and the amount remaining in the cage after 24 h. The average consumption was determined for each animal. The total kilo-calorie intake was estimated as a function of the daily feed intake. The energy efficiency coefficient (EEC) was calculated, and the impact of caloric intake on weight gain was considered, where EEC = weight gain/total energy intake [weight gain = final body weight − initial body weight] [20].

### 2.5. Oral Glucose Tolerance Test (OGTT)

Male offspring were subjected to OGTT tests at seven and eight weeks of age. For this, they were placed on an overnight fast of eight hours and were orally administered glucose solution at a dose of 2 g/kg fasting body weight. Glucose levels were taken at 0, 15, 30, 60, 90, and 120 min following glucose delivery, using a glucometer (Accu-check active) on blood obtained from each animal’s tail vein. The glucose response curve was plotted, and the area under the curve (AUC) was calculated using a trapezoidal method.

### 2.6. Analysis of Biochemical Parameters

The male offspring of the groups studied were euthanized at nine weeks of age by administering 120 mg/kg of sodium pentobarbital. The blood collected from each animal was centrifuged at 3000× *g* at 5 °C for 15 min to obtain the serum. A commercial kit (SPINREACT, Barcelona, Spain) was used to measure the components, including total cholesterol (TC), triglycerides (TG), high-density lipoprotein cholesterol (c-HDL), and high-density lipoprotein cholesterol (c-LDL).

### 2.7. Tissue Weight and Body Fat

After the blood extraction, the visceral and liver adipose tissues were dissected. Subsequently, the organs were weighed (in grams) using an analytical bascule.

### 2.8. DNA Extraction and 16S rRNA Gene Amplification

After the offspring had been dispatched, fecal samples (0.1 g each) were collected using sterile microtubes. DNA extraction of each sample was performed using the Quick-DNA™ Fungal/Bacterial kit (Zymo Research, Irvine, CA, USA). The purity of the DNA was determined spectrophotometrically using a Nanodrop™ (ThermoFisher Scientific ©, Waltham, MA, USA). The integrity of each gDNA sample was checked using 0.7% agarose gel (p/v) electrophoresis. Finally, the samples were stored at −70 °C until use.

The V3–V4 region of the bacterial 16S rRNA gene was amplified by PCR from gDNA samples using the 16S primers 341F: 5′ CCTAYGGGRBGCASCAG 3′ and 806R: 5′ GGACTACNNGGGTATCTAAT 3′. All PCR reactions were carried out with Phusion^®^ High-Fidelity PCR Master Mix (New England Biolabs, Ipswich, MA, USA). The PCR products were analyzed on a 2% agarose gel and purified using a Qiagen Gel Extraction Kit (Qiagen, Hilton, Germany).

### 2.9. Library Preparation and Sequencing

Sequence libraries for sequencing were prepared using the NEBNext© Ultra™ DNA Library Preparation Kit for Illumina (New England Biolabs, Ipswich, MA, USA). Index codes were added to the libraries, and their quality was evaluated using the Agilent Bioanalyzer 2100 system and Qubit@ 2.0 Fluorometer by Thermo Scientific. The Illumina platform was used for sequencing, and it generated 250 bp paired-end reads.

### 2.10. Bioinformatics Analysis

The raw data from the sequencing experiments were processed using the DADA2 package to resolve amplicon sequence variants (ASVs) [21]. We used the following filtering criteria: (i) two error thresholds in sense and antisense reads; (ii) removal of sequences with ambiguous bases; (iii) trimming of the first 18 bases in the sense reads and 12 bases in the antisense reads; and (iv) the sense reads were truncated at a length of 280 bases and the antisense reads at a length of 265 bases. From the filtered sequences, error modeling was performed [21,22], and surviving sequences were merged and filtered in order to remove chimeric sequences using the “*removeBimeraDenovo*” algorithm with the “consensus” method [22]. The taxonomic assignment of sequences was performed using the Bayesian classification method [23] and SILVA database version 138 [24]; the phyloseq object with the Phyloseq package [25]; and the phyloseq object was transformed into a MPSE object with the MicrobiotaProcess package [26] to estimate the rarefaction curve with the *mp_cal_rarecurve* function. The phyloseq object was transformed into an R6 object with the microeco package [27], and it was used to estimate and compare the Shannon alpha diversity index using a Wilcoxon test with a significance threshold of 0.05. To identify the differential signature gut microbiota at the genus level, we used a machine-learning algorithm (Random Forest) provided by the microeco package [27,28] LDA and a *p*-value cutoff of 1 and 0.05, respectively. This algorithm was also used to identify differentially abundant taxa at the genus level and test how well a model could classify samples for each treatment group. The fifteen most important genus-level indicators (determined by the mean decrease GINI score) used by the models to classify samples by group were selected using a *p*-value cutoff of 0.05. Plots were made with ggplot2 functions [29], and all analyses were done in the Rstudio software 2024.09.0+375 [30].

### 2.11. Statistical Analysis

The assumptions of normality were tested with the Shapiro–Wilk test, and homogeneity was tested with the Fligner and Levene tests. Accordingly, ANOVA of one factor, or Kruskal–Wallis, was applied, considering a *p*-value cutoff of <0.05. Principal component analysis (PCA) was used to summarize and visualize the information about the weight of mothers and offspring among the experimental groups. For this, the weight of each group collected along the time course of the experiment was transformed with the prcomp function, and the results were plotted with the autoplot function within the ggfortify package [28]. Statistical analyses were performed in the Rstudio software 2024.09.0+375 [30].

## 3. Results

### 3.1. Effects of L. rhamnosus GG on Food Consumption and Body Weight of the Offspring

**Figure 1:**
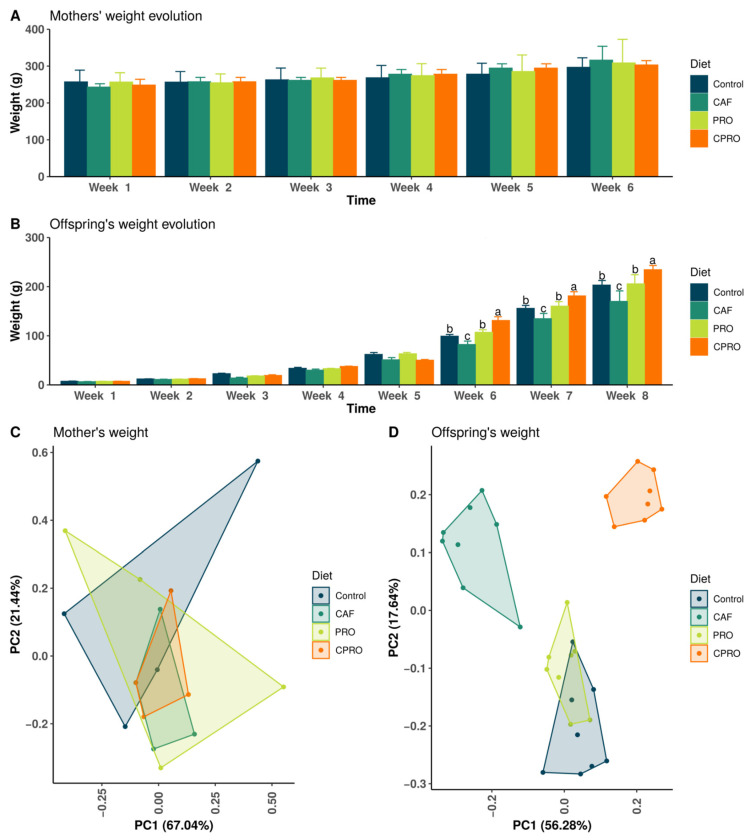
Comparison of the results obtained from the evolution of the maternal body weight and the offspring. (**A**) Body weights of mothers (g) during the nine weeks of maternal programming (*n* = 4/group), (**B**) weights of offspring up to the eighth week of age (*n* = 8/group), (**C**) PCA of mother’s weight (*n* = 4/group), and (**D**) PCA of offspring’s weight (*n* = 8/group). Values in the superscript with different letters are statistically significant (*p* < 0.05). CAF = cafeteria group, PRO = probiotic group and standard diet, and CPRO = cafeteria diet and probiotic group.

At the beginning of the study, the female Wistar rats had similar average body weights (approximately 240 g) and were under the same breeding conditions. This trend remained until the end of the maternal programming period, as no statistically significant differences were observed in the maternal weight among treatments (Table 1, Figure 1A). Female rats fed with the hypercaloric formula and probiotic supplementation (CPRO group) significantly increased their food intake (about 25.6 g) compared with the other groups. The mothers of the CPRO group also presented a higher daily kcal intake and a lower energy efficiency coefficient, statistically significant with *p* < 0.001, compared with the control and PRO groups (Table 1).

Table 1 shows that the male offspring of the maternal programming by the cafeteria diet (CAF) presented low birth weights with lower weight gains during postnatal development (*p* < 0.05). Meanwhile, the offspring of mothers exposed to the cafeteria diet with probiotic supplementation (CPRO) presented average birth weights and postnatal weight gains from 6 weeks of age (Figure 1B), with reduced food intake (11.1 g/day^−1^) and kcal levels and higher EEC values compared with the other groups (*p* < 0.05). These results coincide with those obtained with the PCA (Figure 1). In the PCA of the mother’s weight (Figure 1C), the data are distributed in the same vector space regardless of the experimental group considered. At the same time, in the case of the offspring (Figure 1D), it is noticeable that the PRO and control groups are independent of the CPRO and CAF, which separate to form distinct groups from the rest.

### 3.2. The Effects of L. rhamnosus GG Supplementation on OGTT in the Offspring of the Experimental Groups

**Figure 2:**
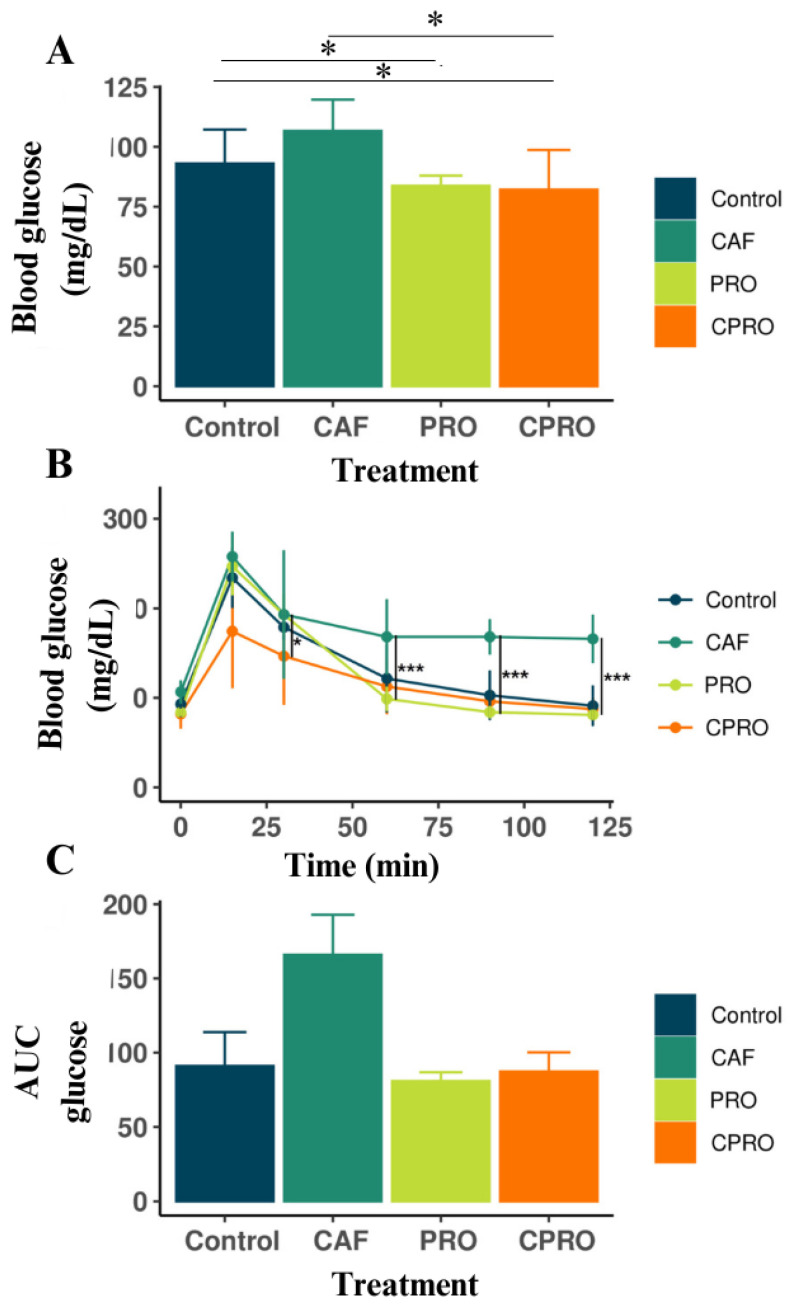
Comparison of the glucose tolerance test results among the descendants of each analyzed group. *, ***, Values are statistically significant (*p* < 0.05), (**A**) Basal glucose levels (mg/dL). (**B**) Oral glucose tolerance curve (mmol/L). (**C**) The area below the AUC curve. CAF = cafeteria group, PRO = probiotic group and standard diet, and CPRO = cafeteria diet and probiotic group. (*n* = 8/group).

We assessed the glucose tolerance of male offspring at seven weeks of age to examine the impact of the cafeteria diet and *L. rhamnosus* supplementation on glucose sensitivity. We found significant differences in the basal glucose levels among the treatments (Figure 2A). Figure 2B shows that the offspring of mothers exposed to the cafeteria diet (CAF) presented a significant alteration in glucose sensitivity compared with the other groups (*p* < 0.001). The progeny of the CPRO group displayed normal basal glucose levels and glucose sensitivity during 120 min of OGTT. The CAF group offspring had an increase in AUC (Figure 2C), while the CPRO offspring had a reduction compared with the control groups. These data indicate that exposing mothers to a cafeteria diet increases the glucose sensitivity of the offspring, whereas supplementing with *L. rhamnosus* GG during pregnancy helps to decrease it.

### 3.3. Supplementing L. rhamnosus GG During Maternal Programming Mitigates the Effects of the CAF Diet on the Lipid Profile and Weight of Visceral Fat Tissue and Modulates the Gut Microbiota in the Offspring

Table 2 shows that the offspring of mothers supplemented with LGG (CPRO group) presented serum levels of TG, HDL-c, and LDL-c, with decreased visceral adipose tissue weight compared with the CAF group (*p* < 0.05). By contrast, there was no statistically significant difference in the liver weight and TC of the offspring (*p* > 0.05).

**Figure 3:**
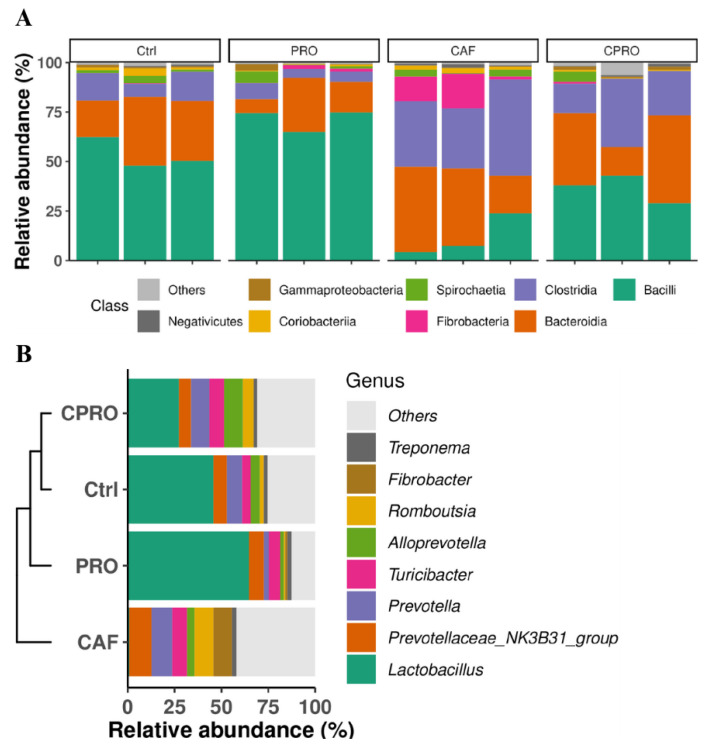
Effect of LGG supplementation on the intestinal microbiota profile of the study offspring. (**A**) Relative abundance at class level. (**B**) Gender-related abundance. Ctrl = control group, CAF = cafeteria group, PRO = probiotic group and standard diet, and CPRO = cafeteria diet and probiotic group.

Maternal supplementation with LGG modulates the intestinal microbiota of offspring. The 16S RNA gene sequencing was analyzed to assess the impact of cafeteria diet intake during pregnancy on the bacterial communities of male descendants. At the class level, we found that the control and probiotic-supplemented groups showed a higher prevalence of the Bacilli class and a significant decrease in Bacteroidia. In contrast, descendants of the CAF group showed a more substantial predominance of Bacteroidia, Clostridia, and Fibrobacteria, with a statistically significant reduction in Bacilli (Figure 3A). At the genus level, the Lactobacillaceae family decreased significantly in the descendants of the CAF group, with a more significant predominance of *Prevotella* and *Prevotellaceae NK3B31,* followed by *Turicibacter, Romboutsia,* and *Fibrobacter* (Figure 3B). Meanwhile, in the offspring of mothers supplemented with the probiotic, there was a reduction in these bacterial genera with an increase in the *Lactobacillus* and *Alloprevotella* genera (Figure 3B).

**Figure 4:**
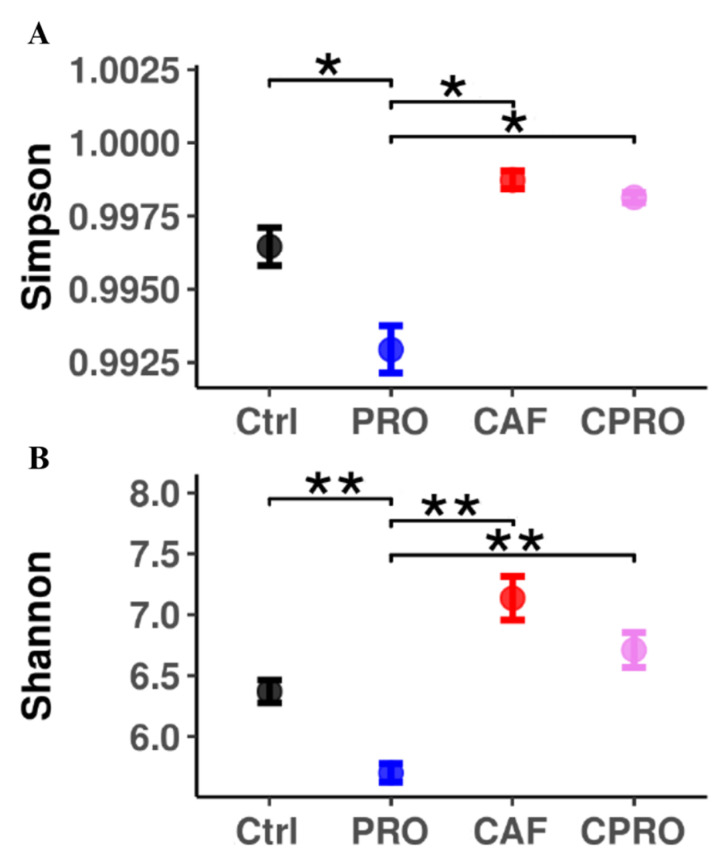
Effect of LGG supplementation on the diversity of the intestinal microbiota profile of male offspring. *, **, Values are statistically significant (*p* < 0.05), (**A**) Simpson index. (**B**) Shannon index. Ctrl = control group, CAF = cafeteria group, PRO = probiotic group and standard diet, and CPRO = cafeteria diet and probiotic group.

**Figure 5:**
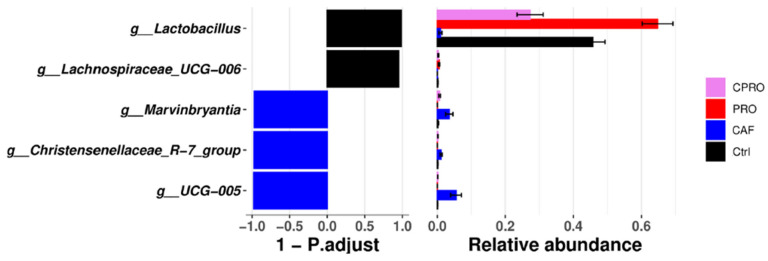
Analysis of the intestinal microbiota based on the average decrease in the Gini index using the Random Forest algorithm and relative abundance. Ctrl = control group, CAF = cafeteria group, PRO = probiotic group and standard diet, and CPRO = cafeteria diet and probiotic group.

The Shannon and Simpson index shows that the alpha diversity of intestinal bacterial communities increases significantly in male offspring of mothers exposed to a CAF diet during maternal programming compared with other groups (Figure 4). According to the differential analysis, we found that the descendants of the CAF group had a lower abundance of the genus *Lactobacillus*, *Christensenellaceae R-7* group (Bacillota), *UCG-005*, and *Marvinbryantia* (Figure 5). In contrast, the male descendants of the control group and the groups of mothers supplemented with the probiotic were enriched in *Lactobacillus* and *Lachnospiraceae UCG-006*.

## 4. Discussion

In recent years, many studies have demonstrated that the nutritional status of mothers plays a crucial role in the long-term health and development of their children during childhood and adulthood [31,32,33]. Consumption of a diet rich in saturated fats and sugars during pregnancy can result in negative metabolic responses and promote complications in the offspring related to fetal adiposity, metabolism, and the immune system [34,35]. Healthy nutrition during pregnancy not only supports the immediate health of the fetus but also forms the foundation for the child’s long-term health [36,37]. Interventions focused on improving maternal nutrition before and during pregnancy are crucial for reducing the risk of disease in future generations [33]. *Lacticaseibacillus rhamnosus* GG is a gram-negative, lactic acid-producing bacteria in the Lactobacillaceae family [38,39]. Studies have shown that taking the probiotic *L. rhamnosus* GG can significantly improve gastrointestinal health, support the immune system, and help prevent or treat conditions such as obesity, diabetes, and insulin resistance [40,41,42].

Our study showed that the offspring of mothers exposed to LGG supplementation had no changes in birth weight and showed an average postnatal weight gain. Meanwhile, offspring exposed to a CAF diet had a lower birth weight and postnatal weight gain. It has been shown that the maternal diet during pregnancy can influence placental function, intrauterine and placental fetal growth during gestation, and the postnatal development of the offspring [43,44]. Inadequate maternal diet can lead to the development of altered biochemical and body markers related to the offspring’s metabolic syndromes [33,45]. However, in our study, these conditions were significantly attenuated by maternal supplementation with probiotics. Supplementation with the probiotic showed potential in regulating the body weight of the offspring, as reflected in the reduction of their visceral fat—results similar to those described in other studies [46,47]. These animals have shown a significant effect on the EEC (*p* < 0.001), suggesting that the probiotic *L. rhamnosus* GG can modulate body weight gain through energy intake [46]. In a previous study, mice fed a high-fat diet (HFD) were supplemented with LGG for 13 weeks, and, as a result, their body weight and fat tissue were reduced. This effect was attributed to the liver’s increased expression of fatty acid oxidative genes, decreased gluconeogenic gene expression, and diminished fatty acid synthesis. The authors propose that probiotic supplementation mitigates lipid accumulation by stimulating adiponectin and activating the AMPK complex [48].

The rise in the consumption of diets rich in fats and sugars is associated with excessive fat accumulation in adipocytes. This, in turn, triggers dysfunction in fat tissue, fostering the development of insulin resistance and alterations in serum markers of glucose homeostasis [47,49]. In this context, our study has demonstrated that maternal programming with *Lacticaseibacillus rhamnosus* GG supplementation improves glucose tolerance and reduces basal glucose levels compared with the control and CAF groups. This effect may be related to the ability of *L. rhamnosus* GG to improve glucose tolerance by relieving endoplasmic reticulum (ER) stress in skeletal muscle, increasing Akt phosphorylation, stimulating GLUT4 (glucose transporter protein) translation, and stopping the activation of inflammatory macrophages (M1) in adipose tissue [50]. In a study using male Wistar rats fed high-calorie or standard diets and administered *L. rhamnosus* GG (viable or heat-inactivated cells), the results indicated that treatments with the active (probiotic) or inactive (postbiotic) strain reversed the increase in basal glucose levels and serum insulin concentration [47]. In this regard, we found that supplementation with *L. rhamnosus* GG during pregnancy significantly decreased serum triglyceride levels in the offspring. This effect can be explained by the ability of the probiotic with *L. rhamnosus* GG to reduce the activation of lipogenic genes and stimulate the suppression of genes responsible for the uptake of long-chain fatty acids. This leads to a significant decrease in serum triglyceride and cholesterol levels [17,48].

Likewise, the intestinal microbiota (IM) is essential in regulating glucose, insulin, and triglycerides related to pathogenesis and treating metabolic diseases such as obesity and diabetes [51,52]. Intestinal dysbiosis promotes an alteration of the intestinal barrier, leading to increased permeability. This allows the translocation of bacterial antigens (e.g., LPS) from the intestinal lumen into the circulatory system, causing an inflammatory response in the host [53]. We analyzed the gut microbiota profile of the offspring and observed that LGG supplementation increased the abundance of bacteria of the genus *Lactobacillus* and reduced the abundance of bacterial groups belonging to the Bacteroidota phylum (*Prevotellaceae, Prevotellaceae NK3B31*) and Bacillota (Clostridia), which were prevalent in the microbiota of offspring maternally programmed with a high-fat diet. The bacteria of the genus *Lactobacillus* play an essential role in normal intestinal homeostasis. Their presence within the intestinal microbiota has been associated with the host’s state of health, and their reduction is linked to diseases such as irritable bowel syndrome and metabolic syndrome [54]. Moreover, LGG is a fermenter of simple carbohydrates and stimulates the secretion of metabolites and proteins with broad-spectrum antimicrobial activity that attack pathogens such as *Escherichia-Shigella, Sutterella*, and *Enterococcus faecalis* [55,56].

The daily supplementation with LGG to rodents, such as probiotics and postbiotics, has modulated their intestinal microbiota, inducing an increase in alpha diversity and bacterial species such as *Akkermansia muciniphila, Blautia glucerasea, Sarcina maxima,* and *L. rhamnosus*, where the interaction between *L. rhamnosus* and *Blautia glucerasea* helped mitigate the alterations of biochemical markers caused by an obesogenic diet [46]. Another study demonstrates that supplementation with LGG and rodent consumption of a high-fat diet showed increased alpha diversity of the intestinal microbiota, with increased Firmicutes and decreased Bacteroidota phylum [50]. In both studies, the animals supplemented with probiotics showed reduced body weight, improved glucose tolerance, and attenuated leptin resistance. These results corroborate our intestinal microbiota profile and biochemical results.

The increased colonization by bacterial species of the Prevotellaceae family (i.e., *Prevotella*) causes a decrease in short-chain fatty acids (e.g., acetate) and IL-18, which leads to the worsening of the inflammatory process, which can cause systemic autoimmune disease [57]. At the same time, the increase in the abundance of bacterial species of the Clostridia class has been associated with inflammatory bowel diseases or antibiotic-associated diarrhea. However, more studies are needed to understand the mechanisms related to the Clostridia class and intestinal microbiota health [58]. We also observed that the descendants of the cafeteria group have presented an increase in the abundance of the Fibrobacteria class. Interestingly, this group of bacteria produces short-chain fatty acids through the fermentation of dietary fibers [59]. They are generally present in the intestinal microbiota of ruminants and hindgut fermenters. Microbial fermentation is essential for maintaining the balance of the intestinal microbiome [59,60]. It is necessary to highlight that the offspring of both groups consumed a standard diet for rodents after weaning and that the presence of this bacterial group could have been acquired from the food. We also observed that male offspring programmed with *L. rhamnosus* GG supplementation had an increased abundance of the *Alloprevotella* genus. This bacterial genus has health benefits, as it produces succinate and acetate, which have anti-inflammatory properties and act to strengthen the intestinal barrier [59,61]. It is important to remember that the intestinal microbiota profile changes and depends mainly on the nutritional and experimental model, explaining some discrepancies in our results.

Overall, our findings corroborate the previously reported beneficial impacts of supplementation with *L. rhamnosus* GG. These results demonstrate that daily administration of the probiotic during maternal exposure to a high-fat diet positively affected the metabolic health of male offspring. This has significant implications for the field of microbiology and nutrition, as it suggests a potential strategy for improving metabolic health. However, the following limitations must be considered. First, our findings cannot be universally generalized to other populations or models, as they are based on a specific strain of rats and particular dietary conditions. Second, it focuses solely on the male progeny, potentially overlooking gender-specific effects.

## 5. Conclusions

Supplementation with *L. rhamnosus* GG during maternal exposure to a high-fat diet improved glucose tolerance and reduced triglyceride, LDL, and visceral fat weight levels in male offspring. Our findings show that maternal exposure to a high-fat diet increased the abundance of the bacterial genera *Clostridia* and *Prevotella* while decreasing the levels of *Lactobacillus*. However, LGG supplementation increased *Lactobacillus* levels and decreased Bacteroidia levels, related to the host’s health status. Our results indicate that supplementation with probiotic bacteria such as *L. rhamnosus* GG during maternal programming (pregnancy and lactation) may protect the offspring from metabolic disturbances caused by a high-fat diet during this critical period. This highlights the importance of maintaining a healthy maternal gut microbiota during offspring development.

## Figures and Tables

**Table 1:** Somatometry and food intake analysis of biochemical markers of male offspring of studied groups.

Parameters	Control	CAF	PRO	CPRO	*p*-Value
**Maternal (*n* = 4/group)**					
Final body weight (g)	279.3 ± 27	323.6 ± 32.7	283.4 ± 55.4	292.4 ± 24.3	0.61
food intake (g/day)	18.8 ± 6	21.3 ± 5.3	21.4 ± 6.6	25.6 ± 6.8 *	**<0.01**
Total food intake (kcal)	62.7 ± 19.8	79.2 ± 22.1 **	71.6 ± 20.7	93.6 ± 28.5 *	**<0.001**
EEC (g/kcal)	1.2 ± 0.8	0.9 ± 0.5	1.2 ± 0.8	0.6 ± 0.3 **	**<0.001**
**Offspring (*n* = 8/group)**					
Weight at birth	7.2 ± 0.2	6.2 ± 0.1 *	6.71 ± 0.2	6.81 ± 0.3	**<0.01**
Final body weight (g)	203 ± 9.6	169 ± 21 **	205 ± 18.7	254 ± 8.0	**<0.001**
food intake (g/day)	18.8 ± 6.5	16.8 ± 6.0	19.6 ± 6.2	11.1 ± 6.5 **	**<0.001**
Total food intake (kcal)	63.3 ± 21.8	62.6 ± 22.5	65.6 ± 20.7	41.2 ± 20.6 **	**<0.001**
EEC (g/kcal)	4.3 ± 1.4	3.5 ± 1.7	3.9 ± 1.2	9.7 ± 5.0 **	**<0.001**

*, **, Values are statistically significant (*p* < 0.05). Data relevance intervals of <0.01 and 0.001 indicate a high level of statistical significance, EEC = energy efficiency coefficient, CAF = cafeteria group, PRO = probiotic group and standard diet group, and CPRO = cafeteria diet and probiotic group.

**Table 2:** Biochemical markers analysis of male offspring of studied groups.

Parameters	Control	CAF	PRO	CPRO	*p*-Value
Visceral fat weight (g)	0.6 ± 0.2	3.4 ± 0.7 ***	0.85 ± 0.6	0.7 ± 0.7	**<0.0001**
Liver weight (g)	14.3 ± 0.9	14.6 ± 3.0	12.2 ± 1.3	13 ± 0.3	0.64
Serum TC (mg/dL)	55.9 ± 6.0	35.3 ± 13.2	35.8 ± 16.2	48.1 ± 10.4	0.81
Serum TG (mg/dL)	34.9 ± 14.0	138.4 ± 30.9 **	41.7 ± 3.9	71.9 ± 16.1	**<0.001**
HDL-c (mg/dL)	45 ± 10.6	25.7 ± 15.0	35.7 ± 9.4	14.4 ± 6.1 **	**<0.001**
LDL-c (mg/dL)	40.2 ± 19.9	132.2 ± 61.8	87.2 ± 20.0	10.3 ± 3.9 ***	**<0.0001**

**, *** Values are statistically significant (*p* < 0.05). Data relevance intervals of <0.01 and 0.001 indicate a high level of statistical significance. Serum TC = total serum cholesterol, Serum TG = total serum triglycerides, HDL-c = high-density lipoprotein-cholesterol, LDL-c = low-density lipoprotein-cholesterol, CAF = cafeteria group, PRO = probiotic group and standard diet, and CPRO = cafeteria diet and probiotic group.

## Data Availability

The data sets obtained and analyzed in the present study are available from the corresponding author upon reasonable request.
